# Gender differences in two-dimensional and three-dimensional speckle tracking echocardiography left ventricular measurements among healthy preschool pediatric population

**DOI:** 10.1186/s43044-023-00380-3

**Published:** 2023-07-05

**Authors:** Ayah Tarek Elsayegh, Hany Nazmi, Hebatallah Mohamed Attia, Heba Kamel

**Affiliations:** Congenital and Structural Heart Disease Unit, Cardiology Department, Ain Shams University Hospital, 38 Abbassia, Next to the Al-Nour Mosque, Cairo, 1181 Egypt

**Keywords:** Two-dimensional global longitudinal strain, Three-dimensional global longitudinal strain, Gender-based difference, Pediatric population

## Abstract

**Background:**

Speckle-tracking echocardiography (STE) is an upcoming echocardiographic modality to measure global as well as segmental left ventricular systolic function expressed numerically as strain values independent of angle and ventricular geometry. We conducted this prospective study on 200 healthy preschool children with structurally normal hearts, to determine gender-based differences in two-dimensional (2D) global longitudinal strain (GLS) and three-dimensional (3D) GLS.

**Results:**

Age-matched 104 males and 96 females were included, 2D GLS results for the males showed longitudinal strain ranging from − 18.1 to − 29.8 with a mean of − 21.7202 ± 5.094322, while for females 2D GLS ranged from − 18.1 to − 30.7 with a mean of − 22.0646 ± 2.167802, also 3D GLS values were measured and compared based on gender, where 3D GLS in males ranged from − 18 to − 24 with a mean value of 20.49 ± 1.28, while for females ranged from − 17 to − 30 with a mean value of 20.47 ± 1.755. The gender-based difference for both 2D GLS and 3D GLS showed non-significant P values.

**Conclusion:**

In healthy subjects below 6 years, 2D STE and 3D STE values showed no difference between males and females, unlike the adult population, to the best of our knowledge, this is one of the few studies in the literature that aims at comparing these measurements in the healthy pediatric group. In routine clinical practice, these values may be used to assess myocardial function or the early signs of malfunction.

## Background

Speckle-tracking echocardiography (STE) is an upcoming echocardiographic modality to measure global as well as segmental left ventricular (LV) systolic function expressed numerically as strain values (LVS) independent of angle and ventricular geometry [14]. Thus enabling early detection of LV dysfunction. 2D-STE is considered a well-established modality for LVS assessment [13]. Recently, 3D-STE has emerged to overcome the technical limitations of 2D-STE. It allows tracking tissues out of the imaging plane. The European Association of Cardiovascular Imaging (EACVI conducted the largest study called the NORRE study, to evaluate the LVS patterns by 2D-STE in healthy adults. Reference values in healthy adults were concluded alongside age and gender-related differences [12].

Up to date, normal measurable ranges for LV systolic functions as assessed by 2D and 3D STE are not reported to be equal for males and females. Few studies have measured and compared systolic left ventricular function (LVF) in the adult population according to gender and results were in favor of women [3, 6]. Andre et al. in 2015 found that men had higher radial strains and lower circumferential and longitudinal strains with less negative values [1]. In a trial to explain these findings, a few mechanisms were highlighted including the effect of female sex hormones on cardiomyocyte increasing calcium influx due to higher density of sarcolemmal calcium [3] or increased activity of the actomyosin ATPase with higher calcium sensitivity of contractile proteins [6].

However, other studies failed to conclude statistically significant differences in LV longitudinal strain between males and females [1].

Knowing the gender-specific values should be used to identify subnormal LV systolic function. [4], hence the importance of establishing and comparing LV systolic functions by 2D and 3D STE in both sexes to be able to determine subclinical LV dysfunction in the pediatric population.

To our knowledge the gender difference among preschool age in LV function by 2D and 3D strain hasn’t been studied previously, accordingly in the current study, our aim was to compare and establish normal reference values for 2D and 3D-STE longitudinal strain values between males and females in a group of healthy preschool children and to highlight the age of transition to the reach the values of the adult pattern.

## Methods

- We conducted this prospective study on 200 healthy preschool children below 6 years, who were referred for elective outpatient transthoracic echocardiography in the congenital and structural heart disease unit.
- *Objectives*: To measure 2D and 3D LV GLS in healthy pediatric populations and determine whether there is a statistically significant gender-based difference.
- We included preschool children with a structurally normal heart, we excluded all children with structural congenital or acquired heart disease, abnormal cardiac rhythms, and subjects with systemic diseases affecting LV function.
- The ethical committee approved the study and informed consent was obtained from the subjects’ guardians.

### Two-dimensional transthoracic echocardiography

All subjects underwent ECG-gated transthoracic echocardiography (TTE), studies were performed by an experienced cardiologist with subjects in the supine position using Vivid E9 echocardiographic scanner (GE Ultrasound, Horten, Norway) with a 4.5-MHz (M5S) or an 8 MHz (6S) matrix transducer depending on body weight.

According to the echocardiography recommendations of the American Society of Echocardiography, routine complete 2D, Doppler, and color Doppler were performed in all accessible windows including parasternal and apical windows with Loop recording and storing of two to three cycles for the off-line analysis using the EchoPAC GE 201 version (Chicago, Illinois, United States). Sedation was given when needed.

2D images and cine loops for four, two, and three-chamber views were acquired in the apical window. When the region of interest's breadth had been manually adjusted as necessary, the program automatically traced the LV epicardial border, then the software generates global and segmental longitudinal strain. As normally the myocardium shortens during systole in the longitudinal direction, the longitudinal strain values are normally expressed as negative values.

From these curves, peak systolic longitudinal strain was recorded for each of the myocardial segments then the software averaged results to obtain the global longitudinal strain (GLS represented as percentages. Negative strain values reflect myocardial shortening, whereas positive strain values reflect thickening or lengthening.

### Three-dimensional speckle tracking echocardiography

For the 3D STE assessment, we included only 180 subjects between 2 and 6 years of age. The 20 subjects below 2 years couldn’t be included because of the poor image quality acquired and the inability to perform analysis on the acquired loops, this was attributed to the breathing artifacts and the lack of breath hold with the relatively higher respiratory rate as well as the large size of the multi-beat acquisition probe in relation to the narrow intercostal spaces.

A commercially available ultrasound system (Vivid E95, GE Healthcare, Milwaukee WI) was used, From the apical position full-volume data sets were acquired with the recommended frame rate.

From the 3D full-volume data sets, the apical 4-chamber was automatically extracted. The ideal apical view was chosen by finding views with the largest LV long-axis dimensions in the data set from the apex and the MV. Automatic reconstruction of the 3D endocardial surface was done and when needed manual adjustments were applied. Subsequently, 3D STE analysis was automatically performed segmenting LV into 16 segments. The software provided segmental and peak global longitudinal strain. Consequently, data were recorded including LVED volume, LVES volume, EF, LV mass, stroke volume (SV), cardiac output (COP), and GLS.

### Reproducibility

We randomly chose twenty subjects from our study population to assess inter- and intra-observer agreement of 2D and 3D strain analysis for GLS. For the inter-observer variability, the same data set was examined by 2 different operators 24 h apart, as for intra-observer variability, the analysis was performed two times by the first operator within the time period of one week.

### Statistical analysis

Results were analyzed using Software of the Statistical Package for the Social Sciences (SPSS version 25.0; IBM corp., Armonk, New York, USA).

While categorical data were reported as frequencies and percentages, continuous variables were expressed as the mean and standard deviation. The two-tailed t-test for independent samples was used to assess differences between genders in continuous variables.

## Results

**Fig. 1:**
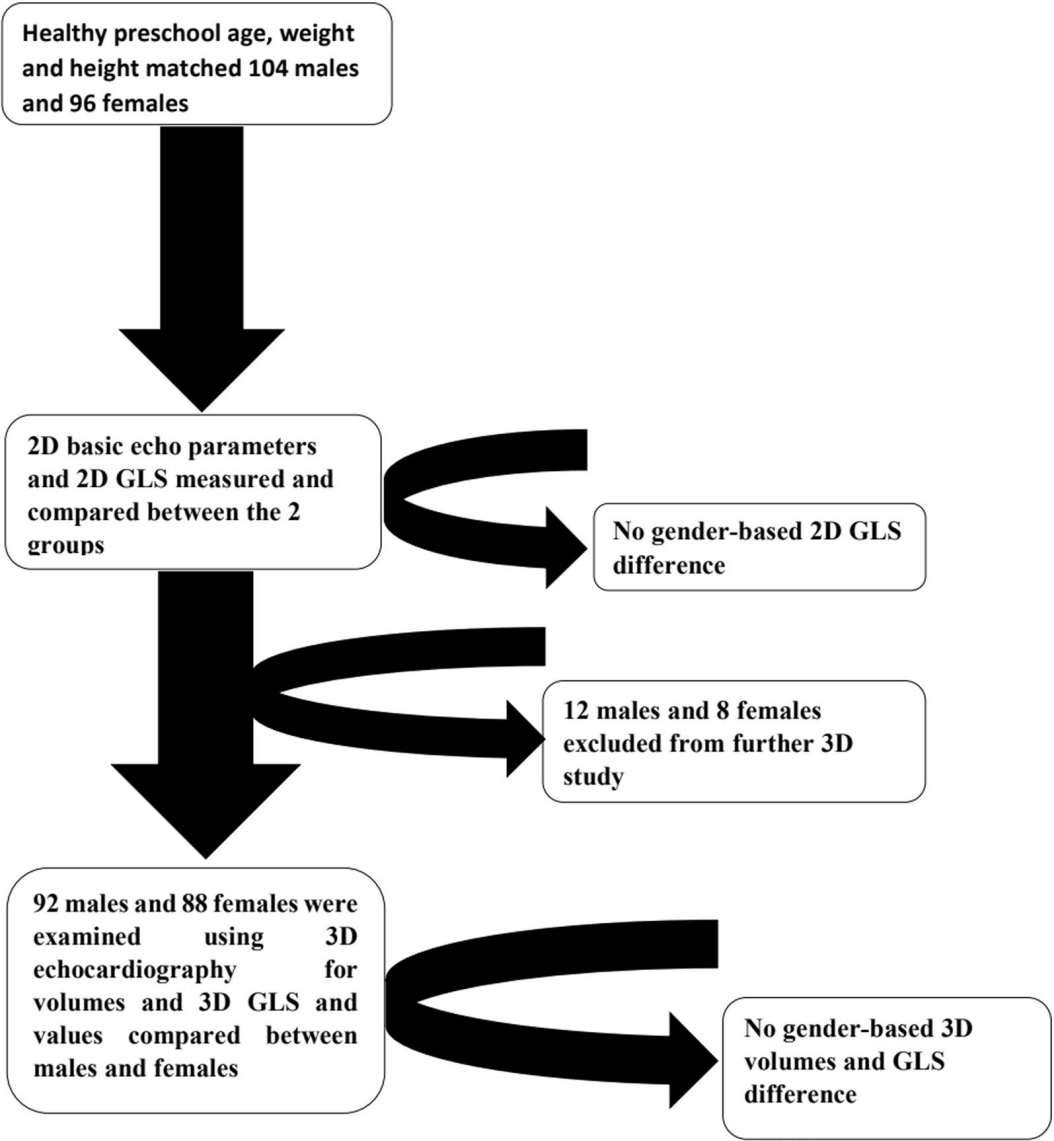
Graphical diagram showing the study population examined by two-dimensional and three-dimensional echocardiography, revealing no gender-based difference in 2D or 3D LV GLS. *2D* two-dimensional, *3D* three-dimensional, *LV GLS* left ventricular global longitudinal strain

Our study included 200 subjects, 104 were males representing 52% of the study subjects, and 96 were females representing 48%. The 2 groups were matched for age, weight, body height, and BSA. 2D STE was performed on the whole study population showing no gender difference in LV 2D GLS values, and 3D STE was performed on 180 subjects, similarly revealing no gender difference in LV 3D GLS, see Fig. 1.

Age for males was 3.7923 ± 1.52565, while for females 3.875 ± 1.523 with a P value of 0.702. P value for weight, height, and BSA was 0.672, 0.916, and 0.627 respectively.

Conventional echocardiographic measurements of the study subjects were recorded and were normal for age without a statistically significant gender-based difference as shown in Table 1.

**Table 1:** Mean values and standard deviation of Conventional echocardiographic measurements

		n	Mean	SD	t	p	Significance
2D.Echo.AO	Males	104	16.56	2.144			
	Females	96	16.67	2.035	− 0.369	0.713	NS
2D.Echo.LA	Males	104	22.73	3.251			
	Females	96	22.9	3.317	− 0.355	0.723	NS
2D.Echo.IVSd	Males	104	4.96	1.114			
	Females	96	4.98	1.187	− 0.108	0.914	NS
2D.Echo.IVSs	Males	104	7.33	0.96			
	Females	96	7.31	1.108	0.098	0.922	NS
2D.Echo.LVPWd	Males	104	4.83	1.038			
	Females	96	4.94	1.014	− 0.762	0.447	NS
2D.Echo.LVPWs	Males	104	7.52	1.07			
	Females	96	7.42	1.194	0.638	0.524	NS
2D.Echo.LVIDd	Males	104	37.17	7.178			
	Females	96	36.54	5.933	0.68	0.497	NS
2D.Echo.LVIDs	Males	104	23.4	6.239			
	Females	96	23.25	5.477	0.186	0.853	NS
2D.Echo.EF	Males	104	0.6475	0.04786			
	Females	96	0.6402	0.04365	1.127	0.261	NS
2D.Echo.FS	Males	104	33.02	3.439			
	Females	96	32.4	2.834	1.403	0.162	NS
2D.Echo.TAPSE	Males	104	21.98	2.473			
	Females	96	22.48	2.521	− 1.409	0.16	NS
2D.Echo.E/A	Males	104	1.458	0.181			
	Females	96	1.46	0.1738	− 0.109	0.914	NS
2D.Echo.E/E’	Males	104	4.98	1.254			
	Females	96	5	1.105	− 0.115	0.908	NS

Comparing the results reveals no gender difference

2D GLS values were measured and recorded. Results for the males showed longitudinal strain ranging from − 18.1 to − 29.8 with a mean of − 21.7202 ± 5.094322, while for females 2D GLS ranged from − 18.1 to − 30.7 with a mean of − 22.0646 ± 2.167802, also 3D GLS values were measured and compared based on gender, where 3D GLS in males ranged from − 18 to − 24 with a mean value of 20.49 ± 1.28, while for females ranged from − 17 to − 30 with a mean value of 20.47 ± 1.755The gender-based difference for both 2D GLS and 3D GLS showed non-significant P values shown in Table 2.

**Table 2:** Mean values, and standard deviation of 2D and 3D GLS strain in both genders

		N	Mean	SD	t	P	Significance
2D.ST.GLS	Males	104	− 21.7202	5.094322			
	Females	96	− 22.0646	2.167802	0.63	0.529	NS
3D.ST.GLS	Males	92	− 20.49	1.28			
	Females	88	− 20.47	1.755	− 0.101	0.92	NS

Comparing values shows no gender-based significant difference

LV volumes and masses derived from 3D full volumes also showed no gender-based significant difference as listed in Table 3

**Table 3:** Mean values and standard deviation for 3D-derived LV volumes and masses in both genders

		N	Mean	SD	t	p	Significance
3D.ST.EDV	Males	92	51.24	12.707			
	Females	88	51.93	11.337	− 0.386	0.7	NS
3D.ST.EDV/BSA	Males	92	72.025	22.37878			
	Females	88	75.2614	21.21499	− 0.996	0.321	NS
3D.ST.ESV	Males	92	23.28	6.997			
	Females	88	23.45	6.434	− 0.172	0.864	NS
3D.ST.ESV/BSA	Males	92	32.4554	10.82976			
	Females	88	35.6795	16.0317	− 1.574	0.118	NS
3D.ST.EF	Males	92	0.6298	0.03245			
	Females	88	0.6225	0.03752	1.39	0.166	NS
3D.ST.SV	Males	92	40.78	4.255			
	Females	88	41.43	3.856	− 1.073	0.285	NS
3D.ST.COP	males	92	4.22	0.42109			
	Females	88	5.0523	5.84102	− 1.333	0.186	NS

Comparing values shows no gender-based significant difference

**Table 4:** Correlation coefficient and covariance results in interobserver and intraobserver variability revealing strong agreement of measurements

Examiner 1 at 1 week interval						Examiner 2 at 24 h interval					
2D GLS	Examiner 1 at baseline	Corr. Coeff			0.998	2D GLS	Ex1_1	Corr. Coeff			0.992
		p			< 0.001			p			< 0.001
		Covariance			3.209			Covariance			3.097
			95% CI	Lower	0.996				95% CI	Lower	0.984
				Upper	0.999					Upper	0.997
3D GLS	Examiner 1 at baseline	Corr. Coeff			0.968	3D GLS	Ex1_1	Corr. Coeff			0.938
		p			< 0.001			p			< 0.001
		Covariance			2.274			Covariance			2.368
			95% CI	Lower	0.913				95% CI	Lower	0.875
				Upper	1					Upper	0.974

### Interobserver and intra-observer variability

We found sufficient interobserver and intraobserver agreement when measuring GLS both by 2D and 3D STE as shown in Table 4.

## Discussion

Speckle tracking techniques are able to detect early subclinical LV dysfunction in a variety of congenital and acquired heart diseases in children. Longitudinal strain is considered by most studies to be a very sensitive measurement of subendocardial dysfunction [7].

The impact of gender differences on strain values in adults has been studied thoroughly, however, only a few studies reported normal values for 2D and 3D STE-derived myocardial deformation in a healthy pediatric population, and even less reports comparing the reference values between males and females.

Augustine et al. concluded that GLS measured by cardiac magnetic resonance was lower in adult males than females which is concordant with our conclusion however in their study it reached significance with a P value of 0.04 and 0.005 respectively [2].

Andre et al. in 2015 had the same conclusion male healthy subjects showed significantly lower longitudinal strains resulting in less negative values [1].

Another study including 155 healthy subjects aged 20–72 years was conducted by Mutluer et al. [9] and concluded no significant gender-related differences in GLS with 3D-STE strain analysis, which could be hypothesized by the different age groups and the inclusion of markedly older population in their study. While controversy exists regarding the influence of age on the global longitudinal strain, several studies reported a decrease in longitudinal strain with increasing age using 2D STE [11].

Few studies have been done to study normal values for 2D and 3D STE-derived myocardial deformation in healthy pediatric populations and even fewer studies to compare the reference values between males and females at this age, although many researchers studied this gender-based comparison in the adult age group establishing normal values and references.

Our study is one of the largest reports of 2D and 3D STE in the pediatric population to date and one of the few to discuss gender-related differences. It is a single-center study that aims to determine gender-specific normal values for LV systolic function in a healthy pediatric population using 2D and 3D STE conducted on 200 subjects.

In our study mean 2D STE GLS values in males and females were similar with no statistically significant difference. In males, values ranged from − 29.8 to − 18.1% with a mean of − 22.2 ± 2.17% while in females it ranged from − 30.7 to − 18.1 with mean values of − 22.06 + 2.17 (P value of 0.662).

In 3D STE mean GLS, the same was evident with no statistically significant difference between the 2 genders. Male values ranged from − 24 to − 18 with a mean of − 20.49% ± 1.28 and for females mean value of − 20.47 ± 1.755 (P value of 0.919).

In 2013 LI Zhang and colleagues studied 228 children in five different age strata (using 3DSTE) There were no statistical differences between both genders for all 3D strain parameters [8].

Most recently, a study conducted in 2022 on 100 healthy normal children to conclude normal reference values for age-dependent GLS values in children and determine its relation to conventional echocardiographic parameters also showed no gender differences in both ventricles GLS [5].

Large-scale research has been done on the differences in heart functioning between the sexes in both animals and humans, however, the studies on males prevail. There is mounting evidence that biological sex affects cardiac health, as well as the development and course of cardiac disease.

There is no considerable variation in cardiac size between males and females till the onset of puberty. This proves that the number of cardiac muscle cells is the same in both genders [10].

All participants in our study population are under the age of six and prepubertal, which means that they lack the metabolic effects of sex hormones on myocardial mechanics and force of contraction. As a result, the lack of statistical significance in our study population may be explained by the age group involved, necessitating further research to determine the age at which gender differences become significant.

## Conclusions

In healthy subjects below 6 years, 2D STE and 3D STE values showed no difference between males and females, unlike the adult population, to the best of our knowledge, this is one of the few studies in the literature that aims at comparing these measurements in the healthy pediatric group.

These values may be used for the evaluation of myocardial function or early onset of dysfunction in a clinical routine setting.

### Limitations

Further studies are still needed including the older age group to be able to accurately determine the timing and mechanism of sex-related differences in echocardiographic measurements that are seen in the adult population. Other studies with the single beat probe will be able to obtain better results on age groups below 2 years of age. Further validation of our results could be done by future studies to include a larger number of subjects.

## Data Availability

All data generated or analysed during this study are included in this published article.

## Abbreviations

BSA: Body surface area
COP: Cardiac output
CI: Confidence interval
EF: Ejection fraction
EDV: End-diastolic volume
ESV: End-systolic volume
FS: Fractional shortening
GLS: Global longitudinal strain
GS: Global strain
LA: Left atrium
LV: Left ventricle
LVF: Left ventricular function
M-mode: Motion mode
MV: Mitral valve
PM: Papillary muscle
PW: Pulse wave
RT3DE: Real-time three-dimensional echocardiography
STE: Speckle tracking echocardiography
SV: Stroke volume
TAPSE: Tricuspid annular plane systolic excursion
TDI: Tissue Doppler imaging
TTE: Trans-thoracic echocardiography
2D: Two-dimensional
3D: Three-dimensional
3DE: Three-dimensional echocardiography
ECG: Electrocardiography
